# The happy learner: Effects of academic boredom, burnout, and engagement

**DOI:** 10.3389/fpsyg.2022.974486

**Published:** 2023-01-10

**Authors:** Christiaan I. Bekker, Sebastiaan Rothmann, Magdalena M. Kloppers

**Affiliations:** ^1^Optentia Research Unit, North-West University, Vanderbijlpark, South Africa; ^2^Research Unit Self-directed Learning, North-West University, Potchefstroom, South Africa

**Keywords:** academic boredom, English, learners, mathematics, satisfaction with life, secondary school, South Africa, subjective well-being

## Abstract

This study aimed to investigate the impact of demographic and contextual variables on boredom in English and mathematics, and to test structural models of boredom, learner burnout, learner engagement, and life satisfaction. Using a cross-sectional survey design and employing a convenience sampling technique, 544 secondary school learners in the Sedibeng District, Gauteng, South Africa, took part in the study. The participants completed the Achievement Emotions Questionnaire – English, the Achievement Emotions Questionnaire – Mathematics, the Schoolwork Engagement Inventory, the School Burnout Inventory, and the Satisfaction with Life Scale. Latent variable modeling was used to test measurement and structural models of boredom, burnout, engagement, and life satisfaction. The indirect effects of boredom on life satisfaction were also computed. The results showed that Afrikaans as the home language, the final mark for English in the previous examination, caregivers that cannot help with English homework, and disliking the English teacher predicted boredom in English. Afrikaans as the home language, marks for mathematics in the previous examination, not having the ability to focus on schoolwork at home, and disliking the mathematics teacher predicted boredom in mathematics. Boredom in mathematics and English resulted in an increase in learner burnout and a decrease in learner engagement. Furthermore, boredom in mathematics and English indirectly affected life satisfaction *via* learner burnout and engagement.

## Introduction

The concept of life satisfaction posits that happiness is a product of human thinking and represents the cognitive component of subjective well-being (Pavot and Diener, 2008; Rojas and Veenhoven, 2013). Life satisfaction reflects the degree to which individuals feel they have met their goals (Diener et al., 1985; Pavot and Diener, 2013) and the resources they have to meet the needs within their environment, either positively or negatively affecting their life satisfaction. This study will report on the associations between academic boredom in mathematics and English, burnout, engagement, and life satisfaction of learners and the possible mediating effect of burnout and engagement between academic boredom and life satisfaction.

Academic boredom is a “silent” but complex and non-trivial aspect of achievement emotion that is negatively associated with optimal learning in formal educational settings (Pekrun et al., 2002, 2010; Pekrun, 2006; Acee et al., 2010). It can impair learners’ physical and psychological health, which, in turn, can harm their perceptions of their abilities, leading to feelings of inadequacy (Preckel et al., 2010; Malkovsky et al., 2012; Willis, 2014). Research suggests that overall diminished quality of life and low life satisfaction (Barnett and Klitzing, 2006; Nett et al., 2011), learner burnout and learner disengagement, and lower levels of achievement are consequences of academic boredom (Nett et al., 2010; Tze et al., 2014; Weybright et al., 2017; Schwartze et al., 2021). For the purposes of this study, burnout, engagement, and life satisfaction are dimensions of well-being of learners.

## Academic boredom and well-being

### Conceptualization of academic boredom

One of the most contemporary, comprehensive, and integrative approaches to understanding emotions in education is the Control-Value Theory (CVT) of Pekrun (2006). Pekrun (2006) developed the CVT of achievement emotion to analyze the causes and effects of emotions in educational settings. The theory’s propositions include appraisal, emotion, environment, and achievement, where each proposition has a reciprocal relationship. The appraisal propositions consist of the subjective value and subjective control over a situation such as learning, achievement, and academic activities, which can have a profound effect on learners’ boredom experience and can be referred to as control and value appraisals (Pekrun, 2006; Acee et al., 2010; Weinerman and Kenner, 2016). Control appraisal refers to an individual’s experience of a specific achievement emotion when they feel in control over a classroom activity, learning, and achievement, whereas value appraisal concerns the meaning these learners attribute to classroom activities (Pekrun, 2006; Artino et al., 2012).

Within the CVT, reference is made to either positive or negative achievement emotions (emotions linked to achievement activities/outcomes). Positive, pleasant emotions refer to enjoyment, joy, hope, pride, and gratitude. In contrast, negative emotions refer to boredom, sadness, anxiety, disappointment, and hopelessness (Pekrun et al., 2007). Depending on the level of control and significance ascribed to classroom activity, the learners will experience different emotions, ranging from pleasure and curiosity to anxiety and boredom (Lichtenfeld et al., 2022). The same principles apply to value; if the activities they participate in have incentive value and are perceived as important, the learner will enjoy learning what is being taught. Conversely, a lack of value and control will lead to boredom (Pekrun, 2006; Pekrun et al., 2010). This implies that control and value appraisals are proximal determinants of these emotions.

Steinberger et al. (2016) defined boredom as a state in which an individual experiences a lack of internal and external stimulation, leading to an active pursuit in the search for something interesting to increase arousal levels and thus alleviate the feeling of being bored. For this study, boredom is defined as a negative, unpleasant achievement-related emotion that refers to an intense and often brief psychophysiological change in response to a supposedly meaningful educational event (Pekrun et al., 2002; Pekrun, 2006; Tze et al., 2013; Westphal et al., 2018). Negative-deactivating emotions like boredom can severely hinder academic learning (Pekrun, 1992; Pekrun et al., 2002). A meta-analysis by Camacho-Morles et al. (2021) showed that boredom is negatively related to academic performance (*ρ* = −0.25).

Highly bored learners typically avoid schoolwork, reduce their efforts in their work, are not well self-regulated, and show reduced motivation (Schwartze et al., 2020). Schwartze et al. (2020) identified positive correlations with behavioral problems, emotional difficulties, and negative affect. Mathematics boredom was also negatively associated with prosocial behavior, positive affect, cognitive reappraisal, and conscientiousness. Brown et al. (2008) showed that boredom was one of the most prevalent reasons learners do not continue with mathematics after secondary school. There is also a negative relationship between boredom and interest (Vogel-Walcutt et al., 2012; Pekrun et al., 2014).

Control-value theory suggests that achievement emotions are governed by universal functional mechanisms. Emotional arousal, therefore, is determined by both control and value appraisals regardless of the academic domain, gender, or cultural background of the learners (Pekrun, 2009). However, recent literature has emphasized the need to investigate cultural and demographic differences in academic boredom proneness (Weybright et al., 2015; Sharp et al., 2016; Lee and Chei, 2020). Prior research indicated that certain cultures were more likely to experience boredom to a higher degree due to sociocultural contexts and internalized cultural values (Tsai et al., 2006). For example, Sundberg et al. (1991) saw Asians as more boredom prone than Westerners. Tze et al. (2013) also reported on cultural influences on appraisals and found that Japanese learners attributed the failure to themselves more than American learners.

It has been found that learners who speak English at home sometimes perform better than those who do not (Howie, 2003; Spaull, 2013). In the research, almost 70% of the pupils answered these tests, set in English, in their second or third language. On the one hand, mathematics performance in wealthier schools is negatively related to repeating a grade once, extra classes, and whether the learner is an orphan. In wealthier communities, a range of human and material resources in schools and homes which enhance and enrich school learning is available (Kotze and Strauss, 2006; Visser et al., 2015). On the other hand, low levels of grade repetition (two or more times) are negatively associated with performance in poorer schools and attending schools in urban areas (Spaull, 2013). Similarly, inconsistencies were noticed in the age and gender differences in the tendency to experience boredom; for example, Wegner et al. (2006) and Daschmann et al. (2011) found that boredom was more common among females and young people, but Hendricks (2015) noticed no gender differences. Goetz et al. (2010) reported higher mean levels of boredom in mathematics and German among Grade 8 learners compared to Grade 11 learners. They also did not find any statistical difference in their mean level of boredom in English.

First-year female students in a South African sample were more likely to report boredom than males; however, no statistically significant correlation was found between boredom and home language (Erasmus and Hall, 2019). Those learners who rated their English language proficiency as good to very good experienced higher levels of boredom (Erasmus and Hall, 2019). Those learners who attended township schools experienced more boredom than those who attended urban schools (Erasmus and Hall, 2019), supporting previous findings that urban areas achieve better mathematics results (Howie, 2003).

Grades did not predict school learners’ boredom. However, prior grades of college students predicted their boredom (Goetz et al., 2007; Schukajlow and Rakoczy, 2016). It is also true that boredom happens when learners are under- or over-challenged (Pekrun, 2006; Schwartze et al., 2020). Demographic variables that influence performance in mathematics in Zambian and Nigerian studies were, e.g., parental education and occupation, parental pressure, learner aspiration and attitudes toward mathematics, enjoyment of mathematics, reading ability, gender, age, and time spent on homework (Georgewill, 1990; Sayers, 1994; Howie, 2003). In schools in more urban areas, pupils who spoke English or Afrikaans at home scored higher in mathematics.

### Learner burnout and engagement

Burnout is a term used to describe a three-dimensional phenomenon involving: (a) exhaustion (a constant feeling of being tired or ruminating on school-related problems due to school demands or pressure); (b) cynicism (an indifferent feeling or attitude toward school or learning); and (c) a sense of inadequacy (a diminished feeling of competency, achievement, or the inability to see things as meaningful) (Maslach et al., 2001; Walburg, 2014; Salmela-Aro et al., 2016; Evers et al., 2020; Lee et al., 2020). Research on burnout among high school learners showed a high prevalence of risk of mental disorders during adolescence (Salmela-Aro et al., 2008; Walburg, 2014). As reported by Winga et al. (2016), low achievers exhibit greater levels of learner burnout, whereas high achievers display lower levels of burnout.

Salmela-Aro and Upadyaya (2012) defined learner engagement as a positive and fulfilling state of mind related to learning. Learner engagement consists of three dimensions: energy, dedication, and absorption. A high level of energy is associated with vigor, whereas dedication is a positive attitude to learning, and absorption is a state of complete concentration in which learning takes place at a rapid rate and time passes very quickly (Salmela-Aro et al., 2016).

Engagement at school is crucial for learners’ learning, academic development, and well-being (Salmela-Aro and Upadyaya, 2012; Upadyaya and Salmela-Aro, 2013; Petillion and McNeil, 2020). Weybright et al. (2017) argued that boredom in schools had been associated with disengagement. Furthermore, learner engagement has been correlated with higher academic achievement (Carter et al., 2012; Forsblom et al., 2022) as well as improved mental health (Steele and Fullagar, 2009) and lower levels of dropouts (Saeki and Quirk, 2015). Hietajärvi et al. (2020) maintain that engagement should be the main aim of modern pedagogical practices by investigating technology-enhanced engagement practices and mitigating the risk factors from boredom experiences. Recent research in a Finnish sample suggests that learners would be more engaged in learning activities if they could use technology (Halonen et al., 2016; Salmela-Aro et al., 2016). Furthermore, continued disengagement will lead to high levels of learners dropping out of school (Weybright et al., 2017).

### Learner happiness: The role of life satisfaction

Happiness can be divided into two concepts: feeling good, i.e., hedonic well-being, and functioning well, i.e., eudaimonic well-being. (Keyes and Annas, 2009). Feeling good refers to those elements that bring joy and pleasure to one’s life, such as life satisfaction and positive affect (Guse, 2020). Moreover, functioning well focuses on those elements in life that lead to meaning, purpose, optimal functioning, living a life of virtue, and human excellence (Ryan and Deci, 2001; Potgieter and Botha, 2020). Eudaimonic well-being activities can lead to life satisfaction. Two theories have been used to explain the feeling-good approach to happiness (Rojas and Veenhoven, 2013). According to one theory, people compare how life is with what it should be. Hence, happiness is based on socially constructed standards of what constitutes a good life and is indicated by life satisfaction. Another theory suggests that people infer happiness based on how they feel most of the time. According to this theory, happiness is an unreasoned (positive and negative) affective experience rooted in the satisfaction of universal human needs. This study focuses on happiness as life satisfaction (rather than positive and negative affect).

The negative effects of boredom, and higher levels of burnout, and disengagement, could impact a learner’s life satisfaction. Research on emotions’ effects in learning has been well-documented (Pekrun et al., 2002, 2011; Linnenbrink-Garcia et al., 2016; Furlong et al., 2021). Emotional learning experiences impact learners’ subjective well-being, quality of learning, motivation, self-regulated learning, and learning strategies.

Academic self-concept is crucial in learner success and well-being (Marsh, 2006; Eccles, 2009; Marsh et al., 2019; Niepel et al., 2021), as well as a broad range of outcomes such as academic emotions (Arens et al., 2017; Pekrun et al., 2019), and even achievement goals (Dörendahl et al., 2021). When learners experience higher levels of positive emotions, it is generally correlated with higher academic performance, whereas negative emotions are often associated with lower academic performance (Leino et al., 2021; Pekrun et al., 2002, 2011). However, learners who perform well academically might not necessarily experience more subjective well-being (Bücker et al., 2018).

Most people experience relatively stable life satisfaction over time (Luhmann and Intelisano, 2018). Guse (2020) found that age is not statistically significantly associated with life satisfaction. Research in the USA showed no life satisfaction changes between Grade 9 to Grade 12 (Huebner et al., 2004). In contradiction, Meuleners et al. (2003) reported a decline in life satisfaction of Australian adolescents between the ages of 12–16 years. Studies on gender and life satisfaction have been largely inconclusive (Chui and Wong, 2016). Compared to females, a slightly higher sense of life satisfaction has been found in males (Goldbeck et al., 2007; Suldo et al., 2015).

### Current study

South African learners performed significantly poorer than most other countries in the TIMSS and struggle to deal with mathematical problems involving language (Maree et al., 2006). The reformation of the schooling system since the advent of democracy in South Africa, aimed to help learners to acquire skills to be lifelong learners, critical thinkers, and problem solvers (Maree et al., 2006). Despite several changes in the South African curriculum, learners still perform poorly in mathematics which has a negative impact on their future job opportunities. Little research has been done on the determinants of boredom in mathematics and English within the South African schooling system. It is unclear how boredom in mathematics and English influences a learner’s life satisfaction. This study aimed to investigate the antecedents of academic boredom in the subject domains of English and mathematics to assess their effect on secondary school learners’ experience of life satisfaction *via* learner burnout and engagement.

## Materials and methods

### Participants

A purposive sample of 544 learners in Grade 9 and Grade 10 were taken (*n*_Grade 9_ = 255; Grade 10 (*n*_Grade 10_ = 226, *n*_Missing values_ = 63). Table 1 describes the biographical variables of the participants.

To be eligible to participate in this study, the participants had to have both English and mathematics as subjects in public secondary schools in the Sedibeng District in the Gauteng Province, South Africa. Their ages ranged from 14 to 19 (*M* = 15.34, *SD* = 0.83). Participants’ self-reported marks for English and mathematics in the previous year’s examination (November 2020) are reported in Table 1.

**Table 1:** Characteristics of participants (*N* = 544).

Item	Category	Frequency	Percentage
Gender	Female	343	63.1
	Male	188	34.6
	Other	4	0.7
	Missing values	9	1.6
Grade	9	255	46.9
	10	226	41.5
	Missing values	63	11.6
Home language	Afrikaans	229	42.1
	English	14	2.6
	African languages	295	54.2
	Missing values	6	1.1
Second language	Afrikaans	29	5.3
	English	393	72.2
	African languages	98	18.1
	Missing values	24	4.4
English marks in the previous exam (November 2020)	50–59%	148	27.9
	60–69%	158	29.8
	70–79%	149	28.1
	80–89%	69	13.0
	90–100%	7	1.3
Mathematics marks in the previous exam (November 2020)	50–59%	228	44.0
	60–69%	131	25.3
	70–79%	87	16.8
	80–89%	52	10.0
	90–100%	20	3.9

211 of the 229 participants who had Afrikaans as their first language had English as a second language (missing values = 18); 168 of the 295 participants who had an African language as their first language had English as a second language, while 105 had an African language as a second language.

### Measuring instruments

A biographical questionnaire, the Achievement Emotions Questionnaire – English (AEQ-E), the Achievement Emotions Questionnaire – Mathematics (AEQ-M), the Schoolwork Engagement Inventory (SEI), the School Burnout Inventory (SBI), and the Satisfaction with Life Scale (SWLS) were administered in this study.

Data about the demographic and contextual variables of the participants were collected using a *biographical questionnaire*. Items included were: current grade; gender; home language; second language; marks received in the last examination for English; marks received in the last examination for mathematics; parent/caregiver help with English and mathematics homework; whether or not participants had a room of their own at home; whether or not participants had a desk and a chair at home to do their homework at; whether participants could focus on their school work at home; having only one teacher in the subject for the entire year; liking the subject teacher; finding the subject interesting, and failing English or mathematics and the entire year because of it.

Two scales of the *Achievement Emotions Questionnaire* (AEQ; Pekrun et al., 2005, 2011; Goetz et al., 2007) were used to measure academic boredom. The AEQ consists of two measures of boredom, one for English and one for mathematics. Learners reported boredom in English (six items; e.g., “I get bored in English classes”) and mathematics (six items; e.g., “I get bored in mathematics classes”) using a Likert scale, which ranged from 1 (“*strongly disagree*”) to 5 (“*strongly agree*”). Learners rated their feelings during a specific subject’s class, studying for it, and taking the associated tests and exams. Averaging the values was done with high values representing high feelings of boredom at each of the three points. Bekker (2022) demonstrated the construct validity and measurement invariance of the AEQ boredom scales for learners in Grades 9 and 10 (using the same sample that was used in this study). The scales have acceptable internal consistency reliabilities ranging from 0.85 to 0.92 (Pekrun et al., 2002, 2005).

Learner burnout was assessed with the *School Burnout Inventory* (SBI; Salmela-Aro et al., 2009). The SBI comprises nine items. Each item is graded on a 6-point scale, ranging from 1 (*completely disagree*) to 6 (*completely agree*). The SBI consists of three subscales: exhaustion at school (4 items, e.g., “I feel overwhelmed by my schoolwork”); cynicism toward the significance of school (3 items, e.g., “I feel that I’m losing interest in my schoolwork”); and a sense of inadequacy as a learner (2 items, e.g., “I often have feelings of inadequacy in my schoolwork”). The SBI has been widely applied across different age groups since this measuring instrument has been adapted to fit the school context (Tuominen-Soini and Salmela-Aro, 2014; Salmela-Aro and Read, 2017) and provides a good overview of learners’ academic and psychological functioning (Upadyaya and Salmela-Aro, 2013; Salmela-Aro, 2017). Cronbach’s alpha coefficient ranges from 0.88 to 0.90, confirming a positive correlation between the test items. In addition, the SBI showed good concurrent validity.

Learner engagement was assessed using three items of the *Schoolwork Engagement Inventory* (SEI; Salmela-Aro and Upadyaya, 2012). The items were measured using a 7-point scale ranging from 0 (*never*) to 7 (*daily*). Three items are included in each subscale: energy (e.g., “When I study, I feel I’m bursting with energy”); dedication (e.g., “I am enthusiastic about my studies”); and absorption (e.g., “Time flies when I’m studying”). The SEI is specified as a uni-dimensional measurement model (Salmela-Aro and Upadyaya, 2012), indicating a general study-related positive state of mind. The SEI has proven to be a reliable and valid tool in Finnish research (Salmela-Aro, 2017). Based on a sum score of school engagement, the SEI has good psychometric properties, indicated by Cronbach’s alpha coefficient of 0.90.

The *Satisfaction with Life* Scale (SWLS; Diener et al., 1985; Pavot et al., 1991; Pavot and Diener, 1993) has gained considerable popularity in recent years (Guse, 2020). To measure the cognitive judgments of life satisfaction, all learners filled out a 5-item scale that measured their level of life satisfaction. The scale consists of items such as ‘*The conditions of my life are excellent*’ or ‘*If I could live my life over*, *I would change almost nothing*’. The learners were required to indicate whether they agreed or disagreed with each of the five items using a 7-point scale system, ranging from 1 (“*strongly disagree*”) to 7 (“*strongly agree*”). In addition to being used widely in multiple countries, the SWLS has also been used in South African studies by a variety of cultural groups (Pavot et al., 1991; Pavot and Diener, 1993; Temane and Wissing, 2006; Keyes et al., 2008; Patel et al., 2009). It has also been validated in Setswana (Wissing et al., 2010). In addition to scalar invariance of the SWLS concerning gender, both metric and scalar invariances were found when age invariance was measured (Jovanović et al., 2022). Cronbach’s alpha coefficients range between 0.79 and 0.89 (Wissing et al., 2010).

### Research procedure

The researcher ensured that parental permission, voluntary participation, informed consent, and confidentiality were maintained during the study. The Health Research Ethics Committee of the North-West University (NWU-00476-19-A1) and the Gauteng Department of Education Research and Knowledge Management Division (Ref: 2019/259A) approved the study.

Schools were invited to participate, and those that wished to participate granted their goodwill permission before parental consent documents were distributed to them. There was an independent person who gathered the consent of the learners before the researcher began the data collection process in each school. Surveys were administered from July to September 2021. The data was captured by independent experts using Epidata software, and the data were checked to ensure that it was accurate on several versions before analyzing the data.

### Data analysis

The data analyses were performed using three statistical programs, namely IBM SPSS Version 27 (IBM Corp, 2020; JASP Team, 2022), and Mplus 8.7 (Muthén and Muthén, 1998–2021). The measurement and structural models were tested using latent variable modeling (Muthén and Muthén, 1998–2021). In this study, all the variables were continuous. The data was checked for multivariate normality and outliers. A scaling correction factor of 1.16 (indicating deviance from multivariate normality) was obtained when a Maximum Likelihood method with robust standard errors (MLR) in Mplus 8.7 was used. Therefore, further analyses used the MLR estimator. SPSS was used to compute descriptive statistics.

As a measure of model fit, both absolute and incremental fit indices were used (West et al., 2012). We measured the accuracy of fit by applying chi-square statistics, standardized root mean residuals (SRMR), and root mean square error of approximation (RMSEA). RMSEA and SRMR values less than 0.08 indicate that the model fits the data adequately. The Tucker-Lewis index (TLI) and comparative fit index (CFI) were used to determine incremental fit indices. It is recommended that TLI and CFI have a value of 0.90 or greater. Models were compared using the Akaike information criterion (AIC) and the Bayesian information criterion (BIC). The models with the smaller information measures have a better fit. The following guidelines were used for differences between the absolute values of the BIC for two models: 0–2 = weak evidence; 2–6 = positive evidence; 6–10 = strong evidence, and higher than 10 = very strong evidence (Wang and Wang, 2020). Concerning measurement invariance for males and females, our analyses showed that the English and mathematics boredom measures were invariant for learners in this study: (a) Boredom (English) – metric against configural (χ^2^ = 4.44, *df* = 5, *p* = 0.488), scalar against configural (χ^2^ = 17.10, *df* = 22, *p* = 0.758), and scalar against metric (χ^2^ = 12.67, *df* = 17, *p* = 0.758); (b) Boredom (mathematics) – metric against configural (χ^2^ = 2.20, *df* = 5, *p* = 0.820), scalar against configural (χ^2^ = 17.57, *df* = 22, *p* = 0.731), and scalar against metric (χ^2^ = 14.63, *df* = 17, *p* = 0.622.

Scale reliability estimates (ω) were computed using JASP (JASP Team, 2022). Reliability coefficients higher than 0.70 were regarded as acceptable (Nunnally and Bernstein, 1994). The statistical significance was set at *p* < 0.01. Cohen (1988) guidelines were used to determine the effect sizes of correlations and the percentage of variance explained. Correlations of 0.5 are large, 0.3 are moderate, and 0.1 is small. Cohen (1988) pointed out that the value of *R*^2^ can be described as follows: higher than 0.25 – large effect; smaller than 0.25 but higher than 0.09 – medium effect; smaller than 0.09 – small effect.

Two structural models were tested in this study. First, a structural model of boredom in mathematics and English was tested. Second, a structural model of life satisfaction was tested. Using Mplus 8.7, simple mediation analysis (Hayes, 2018) was performed (Muthén and Muthén, 1998–2021).

## Results

### Confirmatory factor analysis

An MLR estimator from Mplus 8.7 was used to test five measurement models using confirmation factor analysis (Muthén and Muthén, 1998–2021). Competing measurement models were evaluated by specifying and testing Model 1, while three other contesting models (models 2–4) were equally specified, with differences from Model 1, and tested to confirm which model fits better.

Model 1 consisted of five first-order latent variables: boredom in mathematics (measured by six items); boredom in English (measured by six items); learner burnout (measured by nine items); learner engagement (measured by three items); and life satisfaction (measured by five items). All latent variables were correlated. In Model 2, boredom was specified with 12 observed variables (rather than six items on boredom in English and six in mathematics). In Model 3, learner burnout and engagement were specified using 12 items (including all nine items that measure burnout and the three items that measure engagement). Finally, Model 4 provided one latent variable (well-being), and 29 observations were loaded onto it. Table 2 demonstrates goodness-of-fit statistics for the four competing measurement models described above.

**Table 2:** Goodness-of-fit statistics of competing measurement models.

Model	χ^2^	df	TLI	CFI	RMSEA [95% CI]	SRMR	AIC	BIC
1a	983.12	367	0.87	0.88	0.06 [0.05, 0.06]	0.06	51334.79	51751.79
1b	908.07	366	0.89	0.90	0.05 [0.05, 0.06]	0.06	51245.65	51666.94
1c	836.21	365	0.90	0.91	0.05 [0.04, 0.05]	0.06	51162.97	51588.57
1d	789.82	364	0.91	0.92	0.05 [0.04, 0.05]	0.06	51110.62	51540.51
1e	763.43	363	0.91	0.92	0.05 [0.04, 0.05]	0.06	51080.06	51514.25
2	2101.70	371	0.64	0.67	0.09 [0.09, 0.10]	0.09	52553.93	52953.73
3	1257.91	371	0.81	0.83	0.07 [0.06, 0.07]	0.08	51646.12	52045.93
4	3024.33	377	0.45	0.49	0.11 [0.11, 0.12]	0.11	53721.54	54095.55

^*^Values significant at the *p* < 0.01 level (two-tailed). χ^2^, chi-square; df, degrees of freedom; TLI, Tucker-Lewis Index; CFI, comparative fit index; RMSEA, root mean square error of approximation; SRMR, standardized root mean square residual; 95% CI = 95% confidence intervals; AIC, Akaike information criterion; BIC, Bayesian information criterion; ABIC, adjusted Bayesian information criterion.

The results in Table 2 show that a χ^2^ value of 763.43 (*df* = 363, *p* < 0.001) was obtained for Model 1. The CFI (0.92) and TLI (0.91) fit indices were satisfactory (>0.90), as was the RMSEA (0.05 [0.04; 0.05]) and SRMR (0.06) indicators (<0.80). As expected, all items loaded on their respective constructs. The standardized regression coefficients were all statistically significant (*p* < 0.001). Akaike and BIC fit indices were used to compare alternative models, with the lowest values indicating the best fit. Model 1 fits the data well and was the most parsimonious of the three alternative models (AIC = 51080.06; BIC = 51514.25). The fit of the hypothesized model was acceptable on all the fit indices.

To improve the fit of the selected model, analysis continued in an exploratory mode. Modification indices (MIs) were studied to identify reasons for misfit in the model. Item AEQM13 (“I cannot concentrate because I am so bored”) showed error covariance in relation to item AEQM12 (MI = 79.26; “I think the mathematics class is boring”). It was therefore decided to re-specify the model to allow AEQM13 to correlate with AEQM12 (Model 1b). Model 1b has the following fit statistics: χ^2^ = 908.07 (*df* = 366, *p* < 0.001), CFI = 0.90, TLI = 0.89, RMSEA = 0.05 [0.05, 0.06], SRMR = 0.06, AIC = 51245.65, BIC = 51666.94.

While Model 1 b’s fit improved significantly (ΔAIC = −89.14, ΔBIC = −84.85), the fit statistics on one fit index was below the recommended guideline. Item AEQE13 (“I cannot concentrate because I am so bored”) showed error covariance (MI = 69.61) in relation to item AEQE12 (“I think the English class is boring”). The model was re-specified allowing these items to correlate. Model 1c fit statistics were: χ^2^ = 836.21 (*df* = 365, *p* < 0.001), CFI = 0.91, TLI = 0.90, RMSEA = 0.05 [0.04, 0.05], SRMR = 0.06, AIC = 51162.97, BIC = 51588.57.

Table 2 shows that the fit of Model 1c improved significantly (ΔAIC = −82.68, ΔBIC = −78.37). The modification indices were studied again to see if a better specified model is not achievable. AEQE14 (“I cannot stay awake”) showed error covariance (MI = 44.48) in relation to item AEQE13 (“I cannot concentrate because I am so bored”). The model was re-specified allowing these items to correlate. The fit statistics for Model 1d were as follows: χ^2^ = 789.82 (*df* = 364, *p* = 0.000), CFI = 0.92, TLI = 0.91, RMSEA = 0.05 [0.04, 0.05], SRMR = 0.06, AIC = 51110.62, BIC = 51540.51.

The fit of Model 1d improved significantly (ΔAIC = −30.56, ΔBIC = −26.26). However, the modification indices were studied again to see if a better-specified model was not achievable. AEQM14 (“I cannot stay awake”) showed error covariance (MI = 27.58) in relation to item AEQM13 (“I cannot concentrate because I am so bored”). The model was re-specified allowing these items to correlate. The fit statistics for Model 1e were as follows: χ^2^ = 763.43 (*df* = 363, *p* = 0.000), CFI = 0.92, TLI = 0.91, RMSEA = 0.05 [0.04, 0.05], SRMR = 0.06, AIC = 51080.06, BIC = 51514.25. The fit of Model 1e was acceptable.

### Descriptive statistics, reliabilities, and correlations

Table 3 presents the descriptive statistics of the instruments, their Omega coefficient and their product–moment correlation coefficients. The findings from Table 3 suggest that the Omega coefficients of each measure were acceptable as they met the threshold of ≥0.70 as satisfactory, with scores ranging between 0.72 and 0.90 (Nunnally and Bernstein, 1994).

**Table 3:** Descriptive statistics, reliability coefficients, and Pearson correlations of the scales (*N* = 544).

Variable	ω	Mean	*SD*	1	2	3	4
Boredom (Mathematics)	0.90	2.08^a^	0.99	–	–	–	–
Boredom (English)	0.90	2.35^a^	1.10	0.45^**^	–	–	–
Burnout	0.79	3.66^c^	1.01	0.45^**^	0.39^**^	–	–
Engagement	0.72	4.64^b^	1.75	−0.46^**^	−0.59^**^	−0.40^**^	–
Life satisfaction	0.77	4.55^b^	1.39	−0.27^**^	−0.27^**^	−0.35^**^	0.44^**^

**p < 0.01; ω, Omega measurement reliability; SD, standard deviation; means and standard deviations of scale scores are shown, but factor scores were used to compute correlations; a: minimum = 1, maximum = 5; b: minimum = 1, maximum = 7; c = minimum = 1, maximum = 6.

The correlations in Table 3 show that boredom in mathematics was positively related to boredom in English (medium effect), and moderately, negatively related to life satisfaction (small effect). Boredom in English was also negatively related to life satisfaction (medium effect). Boredom in mathematics was positively related to learner burnout and negatively related to learner engagement (both medium effects). Boredom in English was positively related to burnout (medium effect), and negatively related to learner engagement (large effect). Lastly, burnout was negatively related to life satisfaction (medium effect), while learner engagement was positively related to life satisfaction (medium effect).

### Testing the structural model of boredom

The final measurement model demonstrated good fit to the data (χ^2^ = 831.04, *df* = 277; *p* < 0.001; CFI = 0.83; TLI = 0.80; RMSEA = 0.07, *p* < 0.001 [0.06, 0.08]; SRMR = 0.11). Each item loaded correctly on its respective construct. The standardized regression coefficients were all statistically significant (*p* < 0.001).

For English and mathematics boredom when considered as dependent variables, the standardized regression coefficients can be found in Table 4.

**Table 4:** Standardized regression coefficients with Boredom in English and Mathematics as dependent variables.

Variable	Estimate	SE	Est/SE	*p*
*Boredom in English ON*
Grade	0.07	0.04	1.62	0.105
Gender	0.07	0.04	1.82	0.069
Language	−0.46	0.05	−8.99	<0.001^**^
Performance: English	−0.08	0.04	−2.02	0.043^*^
Caregiver help	−0.12	0.04	−2.70	0.007^**^
Own room	−0.04	0.05	−0.86	0.389
Having own desk	−0.04	0.04	−0.83	0.408
Focus on schoolwork	−0.06	0.05	−1.29	0.196
One English teacher	−0.00	0.04	−0.12	0.909
Like English teacher	−0.32	0.04	−8.05	<0.001^**^
Fail Year: English	−0.00	0.04	−0.04	0.967
*Boredom in Mathematics ON*
Grade	0.03	0.05	0.63	0.530
Gender	0.05	0.05	0.95	0.344
Language	−0.29	0.05	−5.45	<0.001^**^
Performance: Mathematics	−0.15	0.05	−3.15	0.002^**^
Caregiver help	−0.06	0.05	−1.26	0.209
Own room	−0.01	0.05	−0.26	0.794
Having own desk	−0.01	0.05	−0.18	0.859
Focus on schoolwork	−0.11	0.05	−2.35	0.019^*^
One Mathematics teacher	0.04	0.05	0.88	0.379
Like Mathematics teacher	−0.36	0.05	−7.29	<0.001^**^
Fail Year_Mathematics	−0.04	0.05	−0.74	0.457

SE, standard error; Est/SE, estimate divided by standard error; *p < 0.05; **p < 0.01. Language – Home language; Performance: English = What were the last marks you received for English in the November/December exams?; Performance: Mathematics = What were the last marks you received for Mathematics in the November/December exams?; Caregiver help = Is there a caregiver that helps you with your English homework?; Own Room = Do you have a room of your own?; Having own desk = Do you have a desk and chair to do homework at?; Focus on schoolwork = Can you focus on your school work at home?; One English Teacher = Do you only have one English teacher for the entire year?; One Mathematics Teacher = Do you only have one Mathematics teacher for the entire year?; Fail_Year_Eng = Have you ever failed English and failed the entire year because of it?; Fail_Year_Maths = Have you ever failed Mathematics and failed the entire year because of it?

Table 4 shows that boredom in English is best predicted by four variables, namely, Afrikaans as home language (β = −0.46; *p* < 0.01); the final mark for English in the previous (November 2020) exam (β = −0.08; *p* < 0.05); caregivers that cannot help with English homework (β = −0.12; *p* < 0.01); and disliking the English teacher (β = −0.32; *p* < 0.01). Boredom in mathematics is best predicated by four variables, namely, Afrikaans as home language (β = −0.29; *p* < 0.01); mark for mathematics in the previous (November 2020) exam (β = −0.15; *p* < 0.01); not having the ability to focus on schoolwork at home (β = −0.11; *p* < 0.05); and disliking the mathematics teacher (β = −0.36; *p* < 0.01). The demographic variables and contextual factors predict 28.7% of the variation in boredom in mathematics, but for boredom in English, they predicted 44.4% thereof. In both these instances, the *R*^2^ was higher than 0.25, indicating a large effect.

### Testing the structural model of life satisfaction

Considering the data we collected, the final measurement model produced an acceptable fit to the data (χ^2^ = 768.52, *df* = 364; *p* < 0.001; CFI = 0.92; TLI = 0.91; RMSEA = 0.05, *p* = 0.962 [0.04, 0.05]; SRMR = 0.06). All items loaded as expected on their respective constructs. The standardized regression coefficients were all statistically significant (*p* < 0.001). Table 5 shows the results of the structural model of learner boredom, burnout, engagement, and life satisfaction.

**Table 5:** Standardized regression coefficients with learner burnout, engagement and life satisfaction as dependent variables.

Variable	Estimate	SE	Est/SE	*p*
*Learner burnout ON*
Boredom in Mathematics	0.34	0.06	6.30	<0.001^**^
Boredom in English	0.24	0.06	3.97	<0.001^**^
*Learner engagement ON*
Boredom in Mathematics	−0.24	0.06	−4.42	<0.001^**^
Boredom in English	−0.48	0.05	−8.90	<0.001^**^
*Life satisfaction ON*
Boredom in Mathematics	−0.02	0.07	−0.22	0.827
Boredom in English	0.04	0.07	0.59	0.558
Learner burnout	−0.23	0.07	−3.40	<0.001^**^
Learner engagement	0.37	0.08	4.53	<0.001^**^

SE, standard error; Est/SE, estimate divided by standard error; p, obtained significance value.

*p < 0.05; **p < 0.01.

Table 5 shows that learner burnout is predicted by both boredom in English (β = 0.24; *p* < 0.01) and boredom in mathematics (β = 0.34; *p* < 0.01). As expected, learner engagement has a negative relationship with boredom in mathematics (β = −0.24; *p* < 0.01) as well as boredom in English (β = −0.48; *p* < 0.01). It is clear from Table 5 that there is no direct significant impact on Life satisfaction through boredom in mathematics (β = −0.02; *p* > 0.05) and English (β = 0.04; *p* > 0.05), but the indirect relationship through learner burnout (β = −0.23; *p* < 0.01) and learner engagement (β = 0.37; *p* < 0.01) is evident and significant.

Boredom in mathematics and English predicted 24.9% of the variance of learner burnout. This is just on the verge of being classified as a large effect. Boredom in mathematics and English predicted 39.8% of the variance in learner engagement. This variance has a large effect. Boredom in mathematics and English predicted 22.4% of the variance in life satisfaction.

### Indirect effects

For this study, the suggested procedure by Hayes (2018) to determine how boredom in mathematics and English affected life satisfaction indirectly was applied. A two-sided bias-corrected 95% confidence intervals (CI’s) were constructed by bootstrapping (with 10,000 samples) to evaluate indirect effects.

**Figure 1:**
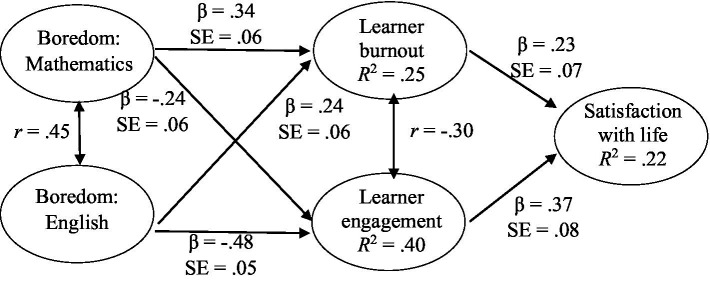
A structural model of life satisfaction.

The results in Figure 1 show that boredom in mathematics (β = −0.08, SE = 0.03, *p* = 0.003, [−0.14, −0.03]), and boredom in English (β = −0.02, SE = 0.02, *p* = 0.016, [−0.11, −0.02]) indirectly affected life satisfaction *via* burnout. The results further showed that low boredom in mathematics (β = −0.09, SE = 0.03, *p* = 0.004, [−0.16, −0.04]), and low boredom in English (β = −0.18, SE = 0.05, *p* < 0.001, [−0.29, −0.10]) indirectly affected life satisfaction *via* learner engagement.

## Discussion

Academic boredom experienced in educational settings is pervasive and has profound consequences for a learner’s burnout, engagement, and life satisfaction (Pekrun et al., 2010). This study aimed to determine the impact of demographic and situational variables on learners’ boredom in English and mathematics. Furthermore, the study aimed to investigate the effects of learners’ boredom in English and mathematics on their happiness, as indicated by their experiences of burnout, engagement, and life satisfaction. This was done considering Pekrun (2006) control-value theory of achievement emotions. Boredom is a negative, deactivating emotion experienced by learners when they take part in educational activities. It was clear from this study that boredom is experienced by learners when they are attending class, doing activities in class, and completing homework activities.

Boredom in mathematics was positively related to boredom in English and negatively related to life satisfaction. Boredom in English was also negatively related to life satisfaction. Corroborating the findings of Barnett and Klitzing (2006) and Nett et al. (2011), boredom in this study was also negatively related to life satisfaction, albeit with small and medium effects. Boredom in mathematics was positively related to learner burnout and negatively related to learner engagement. Boredom in English was positively associated with burnout and negatively associated with learner engagement. Lastly, burnout was negatively related to life satisfaction, while learner engagement was positively related to life satisfaction.

Lower subjective well-being and quality of life were noted by Nett et al. (2010) and Schwartze et al. (2021), also evident from the findings of this study. In support of the findings of this study, Tze et al. (2014) and Weybright et al. (2017) also found academic boredom to be associated with burnout, disengagement, and lower levels of achievement. From this study, learners who disengaged experienced higher levels of burnout. Seeing as adolescents are already at high risk of developing mental health problems or disorders (Salmela-Aro et al., 2008; Walburg, 2014), it would be good to put measures in place to assist teachers and the schools to help alleviate some of the experience of boredom, which influences burnout and engagement, ultimately leading to lower subjective well-being.

Four variables predicted boredom in English: Afrikaans as home language; the final mark for English in the previous examination; caregivers that cannot help with English homework; and disliking the English teacher. Four variables best-predicted boredom in mathematics: Afrikaans as home language; mark for mathematics in the previous exam; not having the ability to focus on schoolwork at home, and disliking the mathematics teacher. No association was found between gender and boredom (see Hendricks, 2015). However, associations exist that point toward an increased experience of boredom when a learner does not have an interest in activities (Daniels et al., 2015; Xie, 2021).

Erasmus and Hall (2019) found no significant correlation between boredom experiences and learners’ home language. However, in this study, the contrary is partially true for one language group. Afrikaans learners were more prone to boredom in English and mathematics than those with English as a home language or an African home language. This confirms the findings of Howie (2003) and Spaull (2013). Also, contradictory to what Goetz et al. (2007) and Schukajlow and Rakoczy (2016) found regarding grades not predicting boredom, this study found that boredom in both English and mathematics are predicted by a previous mark obtained.

Interestingly, caregiver help with homework was statistically significant and negatively associated with English homework, but not with mathematics homework. This might mean that learners believe they can complete mathematics homework independently without needing intervention from caregivers at home. Alternatively, caregiver support with mathematics did not play a significant role because learners might perceive that a caregiver might not be able to help them.

The demographic variables and contextual factors predict 28.7% of the variation in boredom in mathematics, but for boredom in English, they predicted 44.4% thereof. Both these are classified as large effects, meaning that the demographic variables identified in this study, accounted for large percentages of the variance in boredom. Seen in the context of the COVID-19 pandemic, school closures, and in support of Schwartze et al. (2021)‘s findings, the unfortunate environmental circumstances could also lead learners to dislike teachers and their peers, leading to even more maladaptive forms of boredom. Other situational factors could have also played a role, and further research could be done to establish if there are any other situational or personal factors that could influence boredom proneness in secondary school learners. Certain factors such as parental educational level and occupation, pressure, learner aspirations, proficiency in the language, and other contextual factors might have played a role in this, as was found the case in mathematics performance in Zambia and Nigeria (Georgewill, 1990; Sayers, 1994; Howie, 2003).

Also interesting from this study, was the fact that some contextual factors did not impact learners’ experiences of boredom, burnout, or engagement. Demographic variables and contextual factors such as gender; having an own room; having a desk and chair; having one English or mathematics teacher; or previously failing English or mathematics, showed no significance to boredom in either the English or mathematics domain.

Learner burnout was predicted by both boredom in English and boredom in mathematics in this study. As expected, learner engagement had a negative relationship with boredom in mathematics and boredom in English. The experience of boredom has been found to impact learners’ perceptions of their ability, and their sense of adequacy. Therefore, a learner that perceives their own ability to be below average or poor, might not feel competent, and as a result, develop burnout (Malkovsky et al., 2012; Willis, 2014). There is no significant direct impact on life satisfaction through boredom in mathematics and English, but the indirect relationship *via* learner burnout and learner engagement is evident and significant. Learners might be so burned-out that they feel debilitated and end up withdrawing from school or engaging in maladaptive behavior (Shao et al., 2019), thus hampering their academic performance or eventually dropping out of school (Weybright et al., 2017).

Boredom in mathematics and English predicted a large percentage of the variance in learner burnout and an even larger percentage of the variance in learner engagement. Furthermore, boredom and learner burnout and engagement predicted a substantial percentage of the variance in life satisfaction Therefore, boredom in mathematics and English indeed affect the happiness of learners these findings agree with the findings of studies by Tomislava (2021), Botha (2014) and Westaway et al. (2003). Boredom affects learner burnout and has an even stronger effect on their engagement, which affect their life satisfaction, probably because burned-out and disengaged learners do not reach their life goals.

The results showed that boredom in mathematics and boredom in English indirectly affected life satisfaction *via* learner burnout and engagement. As expected, boredom was negatively correlated with academic performance, especially final course grades (Pekrun et al., 2010). Learners experience boredom when they believe a learning activity, or schooling overall, holds no value or significance (Mora, 2011). It, therefore, is important that teachers should note and explain the reasons why activities are to be completed. If learners understand the importance of learning and learning-related activities, it will also contribute to their sense of control (Pekrun, 2006).

Although teachers do their best to make lessons and classes as interesting as possible, some learners will still find those lessons “boring.” Thus, learners need to learn how to create a higher sense of “value” for the mathematics and English subject domains. Learners would like to co-create learning and perhaps allowing learners to choose which activities they could complete would allow them the sense of control they like to have (Xie et al., 2021). In this way, they will target the causes of boredom (Nett et al., 2010). This also allows for happier learners in future.

It remains the responsibility of each teacher and learner, to co-create more stimulating classroom experiences by explaining the relevance of activities or assessments, thereby helping learners understand and value activities more. Fostering a sense of appreciation for lesson content will help the learner understand the value instead of focusing on otherwise boring content (Nett et al., 2011). It will also be good to help learners understand their responsibility in emotional regulation and encourage teachers to be receptive to feedback on lessons, tests, and other educational tools. This will allow the learner to feel more in control, thus enhancing learning outcomes and development (Van Tonder et al., 2022). This will lead to minimizing boredom and maximizing the academic achievement of the learner.

## Limitations and recommendations for future research

There are several limitations to the study. The study’s first limitation was that it was limited to Grade 9 and Grade 10 learners from the Sedibeng District within the Gauteng Province of South Africa. The study needs to be expanded to other provinces. It would be good to include learners from other grades in such a study. The inclusion criteria for participation in this study only looked at boredom in the subject domains of English and mathematics. Further research could also investigate the experience of boredom in other languages, or the domains of economics, business economics, biology and science classes at secondary school levels.

Because this was a cross-sectional study, it is limited that any statements made concerning causality are void of validity as this study only represented a snapshot for this period (Singleton and Straits, 2010). Consequently, future research could also examine a longitudinal approach to determine the cause and effect of these academic emotions. To draw a longitudinal conclusion, it would be beneficial to conduct longitudinal and multilevel studies, which will allow an analysis of the effects of this study’s variables over a longer period rather than at one point in time (Pallant, 2010). Future research could also employ neuroimaging, physiological assessments, and measurements and the analysis of facial expressions and body posture to further examine the effects of boredom. The study relied exclusively on self-reports, which may have contributed to common method variance. In addition, the researcher cannot rule out that having more participants might have affected data analysis and even resulted in different findings, despite the relatively large sample size.

Intervention programs targeting academic boredom in educational settings are still lacking, and research findings like this will have to be validated and tested in various educational settings. Helping learners understand the boredom they experience and helping them cope with such an emotion might be something worth investigating or implementing in future studies. Helping learners understand their emotional experiences better will allow teachers to encourage learners to be more actively involved in the educational experience (Weinerman and Kenner, 2016).

## Conclusion

Academic boredom was classified as an unpleasant, deactivating emotion that should be taken seriously in educational settings. Certain demographic variables and contextual factors play a role in the experience of academic boredom in mathematics and English. These antecedents then have a profound impact on either/both learner burnout and engagement, which impacts a learner’s experience of life satisfaction. This experience creates attention problems and affects motivational engagement and performance in educational settings. Learners who experience academic boredom in either/both mathematics and English have a diminished sense of satisfaction with their lives and might also experience less engagement and higher levels of burnout. Boredom is, therefore, detrimental to secondary school learners’ attention, motivation, efforts, self-directed or -regulated learning, and academic performance.

This study provides valuable information for educators who wish to see happier, more engaged learners in their classes. Results from this study could inform the development of psychoeducational interventions to minimize the effects of academic boredom for these learners within the South African context. Understanding the antecedents that contribute to boredom in subjects such as mathematics and English necessitate teachers to think critically about their teaching strategies and how they structure learning experiences for secondary schools in South Africa. Seeing as academic boredom is a ‘silent’ emotion that sometimes goes undetected, it is even more important that interventions are developed, implemented, and evaluated to at least aim for the reduction of such negative, deactivating emotions, which are detrimental to a learner’s evaluation of their life as satisfied or not.

## Data availability statement

The data is available at Mendeley Data, V1, doi: 10.17632/hscn689xzn.1

## Ethics statement

The studies involving human participants were reviewed and approved by Health Research Ethics Committee, North-West University, South Africa. Written informed consent to participate in this study was provided by the participants’ legal guardian/next of kin.

## Author contributions

CB took the lead in conceptualizing and writing the manuscript and collected and analyzed the data. SR conducted the data analyses, acted as an additional writer, and reviewed the manuscript. MK acted as an additional writer and reviewed the manuscript. All authors contributed to the article and approved the submitted version.

## Funding

This study was supported by National Research Foundation: Grant No: 105850.

## Conflict of interest

The authors declare that the research was conducted in the absence of any commercial or financial relationships that could be construed as a potential conflict of interest.

The handling editor AH declared a past co-authorship with the author SR.

## Publisher’s note

All claims expressed in this article are solely those of the authors and do not necessarily represent those of their affiliated organizations, or those of the publisher, the editors and the reviewers. Any product that may be evaluated in this article, or claim that may be made by its manufacturer, is not guaranteed or endorsed by the publisher.
